# Metabolism of Tryptophan, Glutamine, and Asparagine in Cancer Immunotherapy—Synergism or Mechanism of Resistance?

**DOI:** 10.3390/metabo15030144

**Published:** 2025-02-21

**Authors:** Kajetan Kiełbowski, Estera Bakinowska, Rafał Becht, Andrzej Pawlik

**Affiliations:** 1 Department of Physiology, Pomeranian Medical University, 70-111 Szczecin, Poland; esterabakinowska@gmail.com; 2 Department of Clinical Oncology, Chemotherapy and Cancer Immunotherapy, Pomeranian Medical University, 71-252 Szczecin, Poland; rafal.becht@pum.edu.pl

**Keywords:** cancer, immunotherapy, amino acid metabolism, tryptophan, glutamine, asparagine

## Abstract

Amino acids are crucial components of proteins, key molecules in cellular physiology and homeostasis. However, they are also involved in a variety of other mechanisms, such as energy homeostasis, nitrogen exchange, further synthesis of bioactive compounds, production of nucleotides, or activation of signaling pathways. Moreover, amino acids and their metabolites have immunoregulatory properties, significantly affecting the behavior of immune cells. Immunotherapy is one of the oncological treatment methods that improves cytotoxic properties of one’s own immune system. Thus, enzymes catalyzing amino acid metabolism, together with metabolites themselves, can affect immune antitumor properties and responses to immunotherapy. In this review, we will discuss the involvement of tryptophan, glutamine, and asparagine metabolism in the behavior of immune cells targeted by immunotherapy and summarize results of the most recent investigations on the impact of amino acid metabolites on immunotherapy.

## 1. Introduction

**Figure 1:**
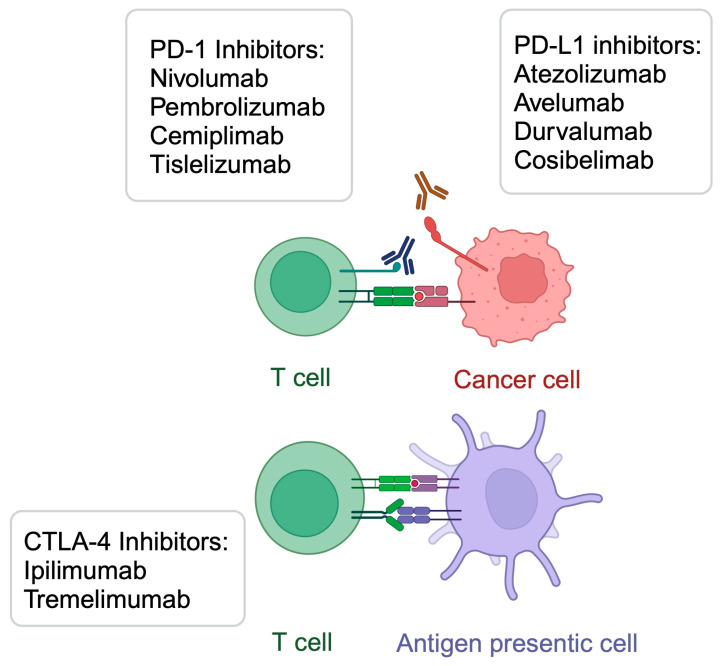
A summary of selected immunotherapeutic agents targeting PD-1/PD-L1 and CTLA-4. Created in BioRender. Kiełbowski, K. (2025) https://BioRender.com/a49x953.

Immunotherapy is an oncological treatment method that induces one’s own immune response to target and kill cancer cells. The discovery of its beneficial effects in the systemic treatment of cancer offered a revolution in oncology, which was awarded with a Nobel Prize in Medicine and Physiology [1]. Immune checkpoint inhibitors (ICIs) utilize monoclonal antibodies that target programmed cell death 1 and its ligand (PD-1/PD-L1) or cytotoxic T lymphocyte-associated antigen-4 (CTLA-4) [2]. A large variety of agents are being used in clinical practice, including pembrolizumab, nivolumab, durvalumab, cemiplimab, tremelimumab, and ipilimumab (Figure 1). Targeting these molecules restores cytotoxic properties of T cells, which increases opportunities to suppress tumor growth. ICIs have demonstrated significant benefits in a variety of neoplasms, including melanoma [3], hepatocellular carcinoma [4], and cutaneous squamous cell cancer, among many others [5]. Immunotherapies other than ICIs involve CAR T cells and cancer vaccines. Despite the breakthrough of immunotherapy, many patients fail to respond to these treatment methods. Analysis of the resistance mechanisms are expected to greatly improve treatment outcomes, by providing insights into introduction of adequate treatment strategies in carefully selected cohorts. Thus far, several mechanisms related to immunotherapy resistance has been already identified, such as cell senescence [6], composition of tumor microenvironment [7,8,9], and alterations in metabolic processes [10], among others. For instance, recent studies highlight the crucial role of lactate metabolism in immunotherapy resistance. Enhanced secretion of lactate, observed in case of mutated lung cancer, is associated with immunosuppressive conditions in tumors directly related to reduced immunotherapy response [11]. Moreover, glucose metabolism has been extensively investigated in the context of immunotherapy. Both glycolysis and tricarboxylic acid cycle (TCA) were correlated with impaired response to immunotherapy [12,13].

Amino acids play a key role in the body’s functionality by way of the building up of proteins. Furthermore, their metabolism frequently involves complex pathways, and the enzymes taking part in these processes, as well as the byproducts of these processes, affect cellular behavior. Dysregulation of amino acid metabolism has been correlated with pathogeneses of numerous diseases [14,15]. Interestingly, accumulating studies demonstrate the involvement of amino acid metabolism in immune system activation. Therefore, that these pathways are considered to also affect the outcomes of cancer immunotherapy. For instance, it has been suggested that aberrant tryptophan (Trp) metabolism is involved in the response to immunotherapy in patients with melanoma [16]. The aim of this review is to summarize recent studies investigating the role of amino acid metabolism cascades in response to immunotherapy.

## 2. Tryptophan

**Figure 2:**
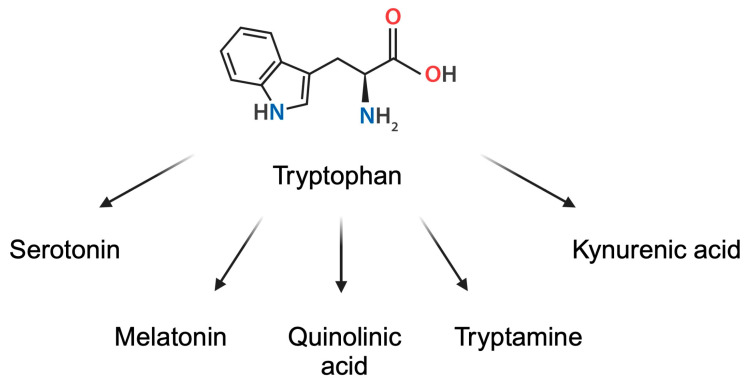
Derivatives of tryptophan. Created in BioRender. Kiełbowski, K. (2025) https://BioRender.com/y78q273.

Being a necessary diet element, Trp is a precursor of several biological compounds, including melatonin, serotonin, quinolinic acid, tryptamine, and kynurenic acid, as well as important coenzymes such as nicotinamide adenine dinucleotide (NAD+) [17] (Figure 2). Importantly, tryptophan is also metabolized by gut microbiome, leading to the production of indoles, compounds responsible for inflammation modulation [18]. Thus, Trp is metabolized towards a variety of biologically active compounds, which significantly affect homeostasis and physiological state.

The kynurenic pathway is considered to be one of the main cascades in Trp metabolism. Indoleamine 2,3-dioxygenase (IDO) 1 and 2 catalyze the formation of kynurenine, which is a ligand of the aryl hydrocarbon receptor (Ahr) [19]. The receptor is located in the cytoplasm, where it is bound to heat shock protein 90 (Hsp90). Interaction with ligand induces dissociation from Hsp90 and nuclear translocation of the receptor, which then regulates gene expression [20].

Trp metabolites have a profound effect on immune system. For instance, gut microbiome-produced indole-3-acetaldehyde (I3AA) is a Trp metabolite enhancing the differentiation of T cells towards the pro-inflammatory Th17 population [21]. Furthermore, kynurenic acid derived from gut microbiota was recently found to enhance the presence of specific subtypes of macrophages strongly associated with encephalomyelitis [22]. Therefore, it is unsurprising that Trp derivatives modulate immune microenvironment of the tumor and antitumor efficacy. Indole-producing bacteria that are present in the microbiome generate Ahr ligands. The use of antibiotics that suppress the viability of these bacteria reduced tumor weight [23]. In animal models of pancreatic cancer, the use of Ahr inhibitors in combination with PD-L1 antibodies significantly enhanced anticancer activity [23]. Therefore, reducing the activation of the Ahr pathway is suggested to promote antitumor effects.

Regarding IDO enzymes, they were found to have an important impact on immune system responses. For instance, it stimulates the polarization of anti-inflammatory M2 macrophages [24]. Several studies have already proven the potential role of IDO1 inhibitors in the treatment of cancer [25]. Combinations of IDO inhibitors with other anticancer agents, as well as the use of novel dual inhibitors, showed benefits in colorectal cancer (CRC) [26,27], pancreatic cancer [28], and hepatocellular carcinoma (HCC) [25], among others [29]. Mechanistically, Cronin et al. [30] demonstrated that kynurenine suppresses T cell proliferation and enhances the presence of reactive oxygen species (ROS). Tetrahydrobiopterin (BH4) could reverse these results, as its administration in tumor-harboring animal models suppressed growth of the lesions. Another mechanism linking kynurenine with the immunosuppressive environment involves NK cells. These cells demonstrate important cytotoxic activity, which is thought to play a crucial role in cancer surveillance. Patients with lower cytotoxic potential in NK cells are at greater risk of developing cancer. Furthermore, worse outcomes were observed in some populations of cancer patients with diminished NK cell functionality. Due to their ability to secrete chemokines and cytokines, they induce an indirect effect on tumor growth as well, by promoting the infiltration of T cells [31]. It was demonstrated that kynurenine could induce NK cell death [32], thus decreasing anticancer properties. In line with these findings, Huang and colleagues [33] showed that ginseng polysaccharides increase the efficacy of PD-1 monoclonal antibody in tumor bearing mice. Mechanistically, the authors showed that these agents reduced the levels of kynurenic acid. Therefore, kynurenine is associated with impaired tumor response.

**Figure 3:**
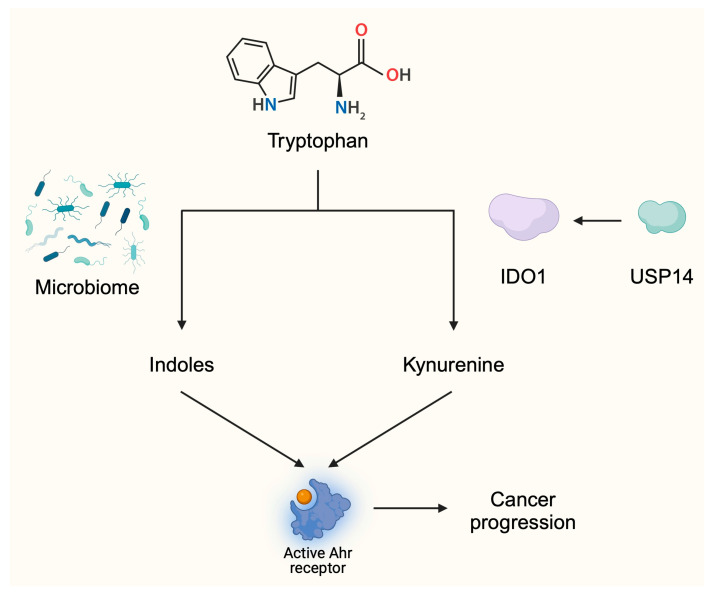
The activity of tryptophan metabolites is associated with tumor progression. Tryptophan can be metabolized into indoles by the microbiome or into kynurenine by IDO enzymes. These ligands activate the Ahr receptor and stimulate pathways and responses associated with cancer progression. These mechanisms provide a background for the development of agents targeting Trp metabolism. Created in BioRender. Kiełbowski, K. (2025) https://BioRender.com/d52f610.

Recent investigations have proved that upstream enhancers of IDO also contribute to impaired anticancer immune responses. One of the promoters of IDO1 expression is interferon γ (IFNγ) [34]. Moreover, stabilization of IDO1 enhances immunosuppressive tumor environment. Specifically, ubiquitin-specific protease-14 (USP14) stabilizes IDO1 expression in CRC cells. Through its deubiquitination, USP14 enhances the expression of IDO1, which impairs immune anticancer effects. Namely, upregulation of USP14 reduced the infiltration of tumor tissue with CD8+ T cells but enhanced it with regulatory T cells (Tregs). In clinical settings, higher expression levels of USP14 have been seen to translate into greater abundances of IDO1 and significantly shorter 5-year survival rates [35] (Figure 3).

As previously mentioned, IDO-targeting agents showed anticancer efficacy in a variety of malignancies. These compounds have especially been examined in the context of cancer immunotherapy. For example, Liang et al. [29] described the use of abrine, an IDO inhibitor, in combination with anti-PD-1 antibodies in Hepa xenograft animal models. Researchers showed that the combination demonstrated synergistic anticancer efficacy. Some of the developed agents entered clinical trials. Additionally, Li and colleagues suggested that suppression of IDO1 activity can be beneficial in tumors resistant to anti-PD-1 therapy [25]. The activity of IDO1 was recently found to mediate the resistance to another mechanism of immunotherapy, namely, the chimeric antigen receptor T cell treatment [36]. Nevertheless, due to failures observed in 2018, stronger distancing arose towards IDO-targeting drugs [37]. These observations highlight the difficulty of translating promising preclinical research into clinical success. Moreover, there is a need to identify a specific cohort of patients that would benefit from such treatments.

**Figure 4:**
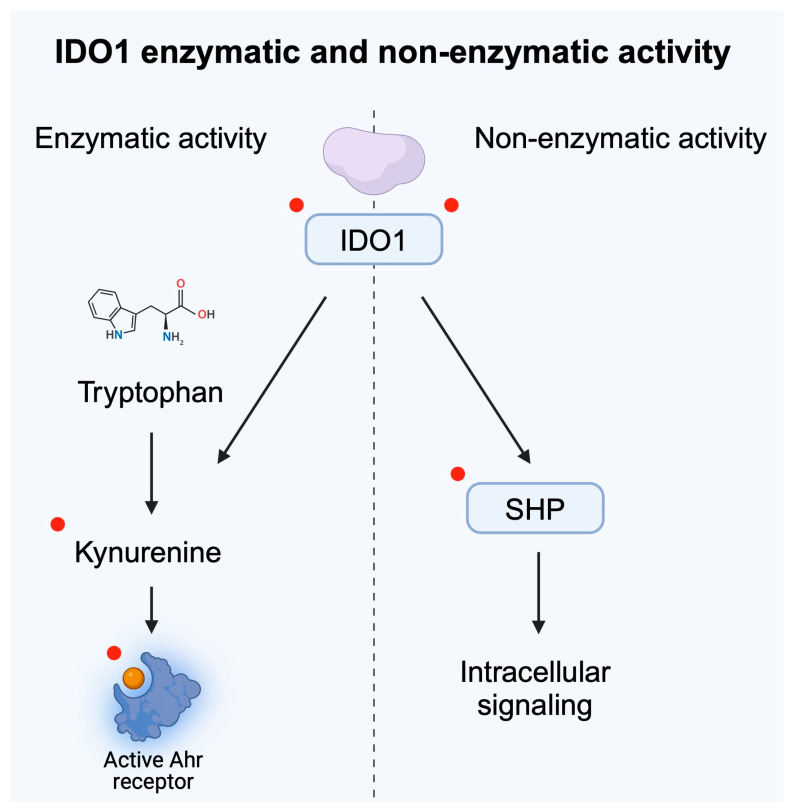
Enzymatic and non-enzymatic activity of IDO1 were both correlated with tumor progression. Red dots highlight potential targets for future cancer treatment and for improving the efficacy of immunotherapy. Created in BioRender. Kiełbowski, K. (2025) https://BioRender.com/z73b504.

Conflicting results were obtained in late-stage clinical trials that examined combinations of IDO inhibitors with pembrolizumab. Epacadostat is an oral IDO1 inhibitor [38] which has been examined in numerous clinical trials. In several types of malignancies, the combination did not show improved response rates compared to the control group [39,40]. In urothelial carcinoma, different results were obtained depending on the included population. In previously untreated cisplatin-ineligible patients with advanced or metastatic urothelial carcinoma, pembrolizumab with epacadostat did not improve response rate [41]. However, in patients who experienced progression after the first line platinum-based chemotherapy, the combination showed antitumor activity, with the overall response rate (ORR) being 26.2%, as compared to the 11.9% observed in the control group [42]. Several potential explanations have been suggested as reasons for not observing a greater clinical efficacy. Therapy incorporating epacadostat was found to insufficiently reduce kynurenine concentrations [43]. Consequently, kynurenine would still promote the immunosuppressive tumor environment, thus limiting the efficacy of immunotherapy. The other potential mechanism includes different functions of IDO1. Despite its enzymatic activity, it also induces intracellular signaling. Structurally, the non-enzymatic IDO1 conformation lacks a heme cofactor. The phosphorylation of tyrosine residues within a non-enzymatic domain makes IDO1 interact with SHP’s family tyrosine phosphatases. As a result of this interaction, IDO1 can bind to the regulatory subunit of PI3K [44]. The non-enzymatic activity of IDO1 enhances tumor progression. In melanoma, IDO1 was found to stimulate the SHP-2/RAS/ERK signaling axis [45], thus contributing to tumor growth. RAS and ERK belong to the family of mitogen activated protein kinases (MAPK) enzymes, strongly implicated in tumorigenesis. Importantly, Panfili et al. [44] showed that epacadostat can enhance the non-enzymatic activity of IDO1. Researchers demonstrated that this stimulation led to a tolerogenic character in dendritic cells (DCs). In support of previous findings, Rossini et al. [46] proved that in ovarian cancer cells, epacadostat increases the presence of non-catalytic IDO1, which enhances tumorigenesis. Therefore, stimulation of the non-enzymatic activity of IDO1 could be the reason why the drug failed in several clinical trials. Recently, another idea emerged regarding the potential suppression of the tumor-enhancing activity of kynurenine. Specifically, application of kynureninase could be used to target and reduce levels of kynurenine, thus reducing immunosuppressive effects of the molecule. Eom and collaborators [47] proved that kynureninase can suppress inhibition of T cell proliferation induced by kynurenine. In addition, researchers showed that the enzyme suppresses tumor growth in in vivo experiments using melanoma and colon cancer animal models. In animal models of non-small cell lung cancer (NSCLC), combination of an SHP-2 inhibitor with anti-PD-L1 agents was associated with more pronounced anticancer immune responses and greater efficacy of radiotherapy [48]. Therefore, current evidence suggests several potential targets that could increase the efficacy of immunotherapy (Figure 4).

Other IDO1 inhibitors were designed as well. However, similarly to epacadostat, some conflicting results were observed. In a preclinical in vitro experiment, stimulation of triple negative breast cancer cells (TNBC) with pro-inflammatory tumor necrosis factor (TNF) cytokine increased cytotoxic properties of indoximod. The drug reduced the viability of cancer cells and enhanced apoptosis [49]. Combination of indoximod with paclitaxel and docetaxel was recently investigated in clinical trial of patients with HER2-negative metastatic breast cancer. However, the majority of included patients demonstrated the presence of a hormone receptor, thus differentiating them from the previously mentioned TNBC cells. This phase 2 clinical trial did not show enhanced efficacy due to the addition of indoximod [50]. On the other hand, preliminary data observed in a phase 1 trial suggests potential efficacy of the drug in pediatric patients with brain tumors [51]. Promising results were achieved in another trial investigating combination of indoximab with pembrolizumab in patients with melanoma [52].

Linrodostat is another oral IDO1 inhibitor [53]. In a clinical study, response to linrodostat was correlated with low tryptophan 2,3-dioxygenase gene expression and high IFN-γ signatures in nonmelanoma patients [54], highlighting the need to identify precise cohorts that will respond to IDO-targeted therapies.

A potential approach is to introduce apo-IDO inhibitors, thus suppressing the non-enzymatic property of IDO1. Perhaps, apo-IDO inhibitors could be combined both with immunotherapy and classic IDO1 antagonists. Such an approach could suppress the total activity of IDO, potentially overcoming failures in clinical trials. Some apo-IDO1 inhibitors have already been developed [55,56,57]. GSK5628, one of the recently developed apo-IDO1 inhibitors, works by competing with heme in binding to heme-free conformation of the enzyme [57]. Importantly, the potential activity of the apo-IDO inhibitor has been explored in preclinical models of cancer. In mouse colon cancer xenografts, a combination of B37 (apo-IDO1 inhibitor) with vascular endothelial growth factor receptor 2 (VEGFR2) inhibitor apatinib induced more extensive anticancer effects than monotherapies [26]. Moreover, the efficacy of B37 combined with immunotherapy was analyzed as well. Liu et al. [58] showed that the use of B37 with anti-PD-1 therapy significantly reduced tumor volume in mice with transplanted colon cancer tumors.

## 3. Glutamine

Glutamine plays a variety of functions in the organism. Its role in pH homeostasis and in exchange of nitrogen represents a crucial step in intermediate metabolism. Furthermore, it takes part in maintaining cellular homeostasis by participating in the development of nicotinamide adenine dinucleotide phosphate, nucleotides, and antioxidants, among others. Glutamine synthetase and glutaminase (GLS) are two enzymes which significantly contribute to the glutamine turnover. The former is typically located in the cytosol; it utilizes energy from the ATP to form glutamine from glutamate and ammonium ions. By contrast, GLS is usually found within mitochondria. It catalyzes glutamine hydrolysis to glutamate [59]. Despite the above-mentioned properties, glutamine, its metabolites and enzymes involved in their formation have immunoregulatory properties. For example, accessibility to glutamine mediates Th1 and Th17 responses [60].

Immunoregulatory properties of glutamine naturally translate into impacts on antitumor efficacy. The use of pan-glutamine inhibitors which target several enzymes involved in glutamine metabolism was shown to modulate the behavior of CD8+ T cells. In 2019, Leone and collaborators [61] proved that suppression of glutamine metabolism reduced growth of several types of animal tumor models. Furthermore, a combination of glutamine suppression with anti-PD-1 treatment significantly enhanced the effectiveness compared to using the immunotherapeutic alone. Researchers analyzed tumor-infiltrating lymphocytes and observed that inhibition of glutamine processing strongly enhanced CD8+ T cell activity and proliferation. Moreover, these cells were also less anergic and less exhausted. Contrary to this, a recent report by Madden et al. [62] showed that the suppression of glutamine metabolism impairs T cell’s ability to target and inhibit tumor growth. The authors propose several possibilities for these conflicting findings. Firstly, the former study utilized in vivo experiments, while the latter was an in vitro study. This notable difference in study design significantly impacts the study environment and conditions. Experiments in animals are associated with different concentrations of active compounds, as well as potential off-target effects on other cells present in tumor microenvironment (TME), thus indirectly shaping CD 8+ T cell responses. Secondly, the authors mention that in the in vivo experiment, the tested drug can modulate the activity of T cells in stages of activation different to those in the in vitro study. Additionally, researchers argue that, depending on drug concentration and inflammatory context, various results may be observed [62]. The involvement of other cells in the TME could indeed affect the observed differences. In a pivotal study, Guo et al. [63] showed that intratumoral injections of glutamine in mice B16 tumor models suppressed tumor growth. Furthermore, this glutamine-based treatment enhanced the response to anti-PD-1 therapy in MC38 tumor models. Mechanistically, researchers showed that these findings are a result of stimulating DCs-mediated CD8+ T cell immunity.

Nevertheless, enhancement of antitumor responses by suppressing glutamine metabolism can be supported through another argument. Specifically, glutaminolysis produces α-ketoglutarate (αKG), a key molecule necessary for the M2 macrophage polarization [64]. The M2 phenotype represents an anti-inflammatory population of macrophages. The use of telaglenastat, a glutaminase inhibitor, was associated with reduced potential of M2 macrophage polarization [65]. Furthermore, αKG is a key element of the citric acid cycle, participating in the generation of ATP, energy molecules strongly needed in malignant cells. Blockage of glutamine metabolism and generation of αKG could reduce energetic needs of cancer cells.

In the context of oncology and immunotherapy, several studies examined the potential benefits of telaglenastat. In mouse models of melanoma, a combination of telaglenastat with either anti-PD-1 or anti-CTLA-4 therapeutics significantly improved the anticancer effect of immunotherapies [66]. Importantly, the drug has been tested in several clinical trials [67,68,69], but to the best of our knowledge, no clinical trial has examined the combination of telaglenastat with immunotherapy so far.

**Figure 5:**
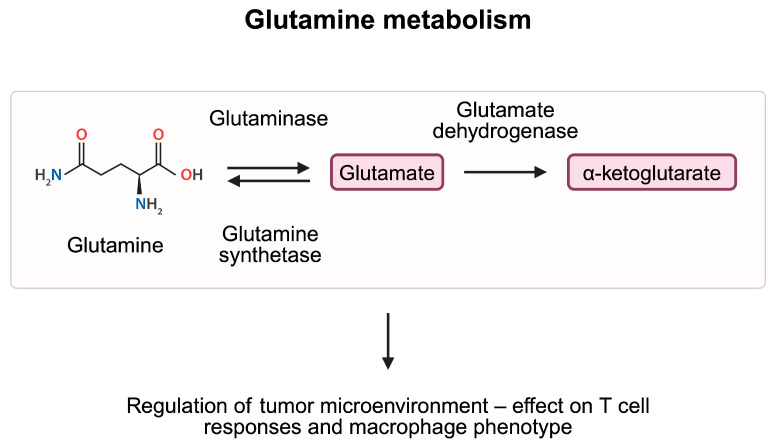
Glutamine is metabolized to glutamate and eventually to α-ketoglutarate, which enters the TCA cycle and takes part in the production of ATP molecules. Studies demonstrated that elements of the glutamine metabolism pathway are involved in the regulation of the tumor microenvironment, thus affecting cancer growth. Created in BioRender. Kiełbowski, K. (2025) https://BioRender.com/z58j607.

Another mechanism that limits glutamine accessibility is suppression of the glutamine transporter. In a review article by Liu et al. [70], the authors nicely summarized the expression of ASCT2 glutamine transporter in various malignancies. V-9302 is a glutamine transporter inhibitor; early studies on this investigated the potential antitumor efficacy of this agent [71]. In a TNBC mice model, V-9302 enhanced apoptosis of cancer cells and restricted tumor growth [72]. Thus, current evidence highlights the involvement of glutamine metabolism in the pathogenesis of cancer (Figure 5).

## 4. Asparagine

Asparagine is a non-essential amino acid, the activity of which, in cancer, has been examined for a number of years. Asparagine is involved in regulation of transport of other amino acids, the activation of signaling pathways, nucleotide synthesis, and cancer progression [73]. Importantly, asparagine significantly affects the behavior of T cells, implicating its involvement in immunotherapy response. The restriction of asparagine shapes responses of T cells differently depending on the cellular activity. Specifically, restrictions during cell activation reduce proliferation. However, stimulation during CD8+ T cell differentiation promotes cellular proliferation. Moreover, reducing the availability of this amino acid potentiates anti-PD-L1 efficacy in mice melanoma models [74]. In a recent study, Zhang et al. [75] describe the starvation-immunotherapy approach to target cancer cells. Researchers developed a method to combine ICIs with a fusion protein involving L-asparaginase and elastin-like polypeptide. This strategy allows for a prolonged and sustained depletion of asparagine in tumor cells, which translates into promising anticancer effects. Nevertheless, the influence of asparagine on T cell activity seems to be more complex. In a series of experiments performed by Wu and colleagues, the authors demonstrated that supplementation of T cells with asparagine enhanced the presence of CD8+ T cells expressing TNFα, IFNγ, and granzyme B. By contrast, the use of asparaginase reversed observed effects. Mechanistically, researchers observed that the effect of asparagine was mediated by the lymphocyte-specific protein tyrosine kinase (LCK), thus demonstrating the involvement of this amino acid in signaling pathways [76]. LCK is a key molecule required for the proper activity of T cells. LCK-deficient animal models demonstrated impaired T cell functionality [77]. In a cohort of bladder cancer patients with muscle invasion, higher expression levels of LCK were associated with better responses to immunotherapy [78]. In line with these findings, Hope and collaborators [79] showed that asparagine contributes to the activation of T cells after the TCR stimulation. Hypothetically, the conflicting results observed regarding asparagine activity could result from different pathways mediated by amino acids, as the relationship between asparagine and LCK is suggested to have beneficial effects.

**Figure 6:**
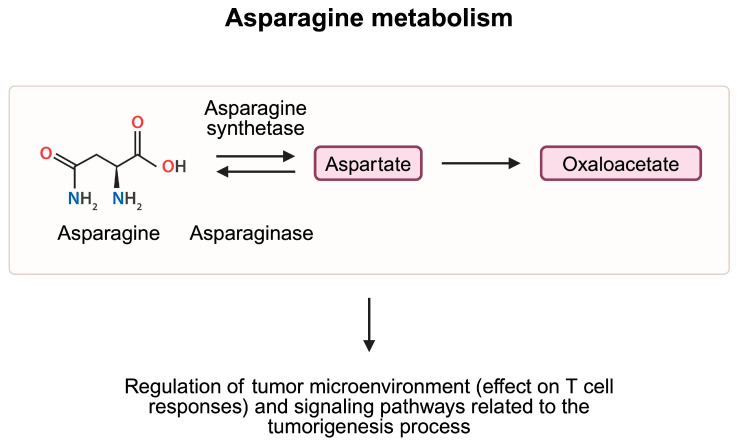
Asparagine is metabolized to aspartate, and eventually to oxaloacetate, which takes part in the production of ATP molecules in the TCA cycle. Studies demonstrated that elements of the asparagine metabolism pathway are involved in the regulation of CD8+ T cell responses and signaling pathways associated with tumorigenesis and autophagy, and thus have a complex role in cancer pathogenesis. Created in BioRender. Kiełbowski, K. (2025) https://BioRender.com/n01h835.

Additionally, studies have identified pathways that influence cancer progression by modulating asparagine metabolism. For instance, in a recent study by Wang et al. [80], the authors demonstrate that the NIMA-related kinase 8 (NEK8) is associated with worse survival and lymph node metastasis in patients with gastric cancer. Further analyses showed that NEK8 expression is positively correlated with that of asparagine synthetase (ASNS). Through phosphorylation of the enzyme by NEK8, ubiquitination and degradation of ASNS is reduced. Therefore, greater activity of the NEK8 kinase leads to greater expression of ASNS, thus increasing the levels of asparagine. The authors also showed that asparagine can activate the mTOR pathway. Therefore, by modulating mTOR activity, asparagine regulates signaling pathways involved in tumorigenesis, but can also mediate the process of autophagy (Figure 6). Another enzyme linked with asparagine metabolism and autophagy is asparagine endopeptidase, which catalyzes the cleavage of asparagine residues. By regulating lysosome homeostasis, enzyme expression was correlated with survival in patients with breast cancer [81]. Moreover, the activity of the enzyme was associated with cancer metastasis [82].

## 5. Conclusions

Amino acids are crucial components of cellular survival and functionality. In addition to building proteins, these molecules demonstrate a variety of different properties and actions, such as taking part in ATP production, pH homeostasis, synthesizing a number of bioactive compounds, stimulating intracellular signaling pathways, and having immunoregulatory mechanisms. In this review, we focused on the involvement of Trp, glutamate, and asparagine metabolism in cancer progression and responses to immunotherapy. Recent studies have demonstrated that metabolites of these amino acids have strong immunoregulatory properties that mediate antitumor activity. Several elements in Trp metabolism have been identified and suggested as potential therapeutic targets, the modulation of which could be combined with immunotherapies. Nevertheless, the testing of currently designed IDO1 inhibitors requires that the patient cohorts who would benefit from the therapy be identified. Future research should continue the current trend of designing inhibitors of the non-enzymatic activity of IDO1. Furthermore, a combination of agents that target both enzymatic and non-enzymatic activities should be investigated. Glutamine demonstrates a variety of activities, including several potential anticancer mechanisms. Targeting enzymes catalyzing glutamine metabolism showed anticancer efficacy and potential synergism with immunotherapy. However, the role of glutamine in TME seems complex and requires further research. Similarly further studies are required to better understand the relationship between asparagine and immune cells’ functionality. Furthermore, more comprehensive investigations into amino acid transporters could help to better characterize immunotherapy resistance. It is possible that intratumoral injections of agents restricting particular pathways in amino acid metabolism or access of cancer cells to amino acids will demonstrate benefits. Additionally, the effects of the microbiome on amino acid and on cancer progression should be further studied. Accumulating knowledge about the role of amino acid metabolism in tumorigenesis, together with more advanced immunotherapy strategies could create novel therapeutic combinations that will hopefully increase clinical benefits.

## Data Availability

No new data were created or analyzed in this study.

## Abbreviations

ICIs	Immune checkpoint inhibitors
PD-1	Programmed cell death 1
CTLA-4	Cytotoxic T lymphocyte-associated antigen-4
Trp	Tryptophan
NAD+	Nicotinamide adenine dinucleotide
IDO	Indoleamine 2,3-dioxygenase
13AA	Indole-3-acetaldehyde
HCC	Hepatocellular carcinoma
ROS	Reactive oxygen species
BH4	Tetrahydrobiopterin
IFNγ	Interferon γ
USP14	Ubiquitin-specific protease-14
Treg	Regulatory T cells
ORR	Overall response rate
MAPK	Mitogen activated protein kinases
NSCLC	Non-small cell lung cancer
TNBC	Triple negative breast cancer
VEGFR2	Vascular endothelial growth factor receptor 2
GLS	Glutaminase
TME	Tumor microenvironment
αKG	α-ketoglutarate
